# Seroepidemiology of Hepatitis A in the Croatian Population

**DOI:** 10.5812/kowsar.1735143X.756

**Published:** 2011-12-20

**Authors:** Tatjana Vilibic-Cavlek, Jasmina Kucinar, Suncanica Ljubin-Sternak, Branko Kolaric

**Affiliations:** 1 Department of Virology, Croatian national Institute of public Health, Zagreb and School of Medicine University of Zagreb, Zagreb, Croatia; 2 Istria County Institute of Public Health, Pula, Croatia; 3 Zagreb County Institute of public Health, Zagreb and School of Medicine University of Rijeka, Rijeka, Croatia

**Keywords:** Hepatitis A Virus, Epidemiology, Seroepidemiologic Studies, Croatia

## Abstract

**Background:**

Hepatitis A virus (HAV) has a worldwide distribution, although this distribution tends to be uneven among geographical regions and population groups. The prevalence of anti-HAV antibodies in the general population varies widely among countries. In Europe, the seroprevalence of HAV is reported to range from 32% to 88%.

**Objectives:**

The aim of this study was to determine the seroprevalence of HAV among the general Croatian population.

**Materials and Methods:**

During a 2-year period (2008-2009), a total of 791 serum samples were tested for the presence of anti-HAV total (IgM+IgG) and anti-HAV IgM antibodies using an automated enzyme-linked fluorescent assay (Mini Vidas; bioMérieux, Marcy l'Etoile, France).

**Results:**

The overall anti-HAV seroprevalence was 41.6%. The observed difference in the seroprevalence rates among male and female patients was not statistically significant (44% vs. 39.6%, P = 0.218). A marked increase in anti-HAV seropositivity with age was observed (P < 0.001). The seroprevalence did not differ significantly between participants residing in rural regions (45.3%) and those residing in urban regions (40.6%, P = 0.292).

**Conclusions:**

Our results corroborate those of seroprevalence studies in other developed countries. More than half of the Croatian population (59.4%) is susceptible to HAV infection. Older age is an important predictor for being anti-HAV positive.

## 1. Background

Hepatitis A virus (HAV) is a significant cause of morbidity in many parts of the world. HAV infections account for 1.5 million cases of hepatitis each year [1]. It has a global, although uneven distribution among geographical regions and population groups. The primary mode of HAV transmission is the fecal-oral route, most frequently person-to-person, or by ingestion of contaminated food or water [2]. The exact prevalence, however, is difficult to estimate because of the high proportion of asymptomatic and anicteric infections. Seroepidemiological studies have shown that the prevalence of anti-HAV antibodies in the general population varies widely among countries, from as low as 13% in the Scandinavian countries to nearly 100% in areas of developing countries, such as parts of Africa, Asia, and South America [3]. In these developing countries, exposure to HAV before the age of 9 is almost universal [4]. In developed countries, transmission shifts to older age groups, and seroprevalence increases during adulthood. In Europe, the seroprevalence of HAV is reported to range from 32% (in Italy and Ukraine) to 88% (in Kosovo) [5][6][7][9][10][11][12]. There are, however, very few published studies on the seroprevalence of HAV in Croatia, and these have been limited to specific population groups [13][14].

## 2. Objectives

The aim of this study was to determine the seroprevalence of HAV among the Croatian general population

## 3. Materials and Methods

**Figure 1:**
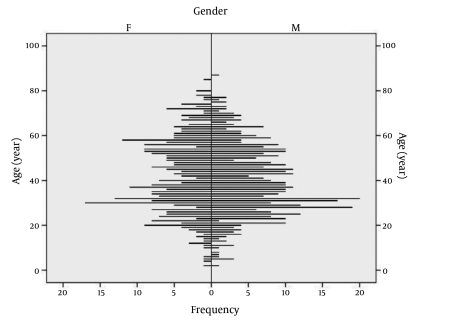
Distribution of Study participants According to Age and Sex

During a 2-year period (2008-2009), a total of 791 serum samples were tested for the presence of anti-HAV total (IgM+IgG) and anti-HAV IgM antibodies at the Laboratory for serologic diagnosis, Croatian National Institute of Public Health and Istria County Institute of Public Health. Serologic tests were performed using an automated enzyme-linked fluorescent assay (Mini Vidas; bioMérieux, Marcy l'Etoile, France). The manufacturer states a diagnostic sensitivity of 99.4% and specificity of 100%. There were 352 (44.5%) males and 439 (55.5%) females aged from 2 to 87 years residing in different cities in four of the 20 Croatian counties (Figure 1). Serum samples were obtained from hospitalized patients: preoperative check-up (cardiac surgery, renal transplant program) and non-hospitalized patients coming for routine testing (physical examination, needle stick injury, patient contacts, lymphatic disorders, antenatal screening, and couples undergoing medically assisted reproduction) with no symptoms of acute hepatitis. The only exclusion criteria were chronic liver diseases. Since no background seroprevalence data was available as a base to calculate sample size, we took the conservative estimate of p = 0.05 and a margin error E = 0.05 (tolerable width of 95% confidence interval of 10%), which gave us a minimum required sample size of 384 examinees. The formula used to calculate the required sample size was n = zα2p(1-p)/E2.

### 3.1. Statistical Analysis

A comparison of categorical variables between groups was made using Fisher's exact test. Statistical analyses were performed using STATA/IC 11.1 for Windows (StataCorp LP, USA). P < 0.05 was considered as statistically significant.

## 4. Results

Of 791 analyzed serum samples,329 (41.6%) were positive for anti-HAV total antibodies. The seroprevalence rate was 44% (155/352) among males and 39.6% (174/439) among females, with no significant difference (P = 0.218). Anti-HAV positivity was low in participants under 30 years of age, ranging from 4.8% to 9.1%. A marked increase in seropositivity with age was observed beginning with the 30- to 39-year age group. The seroprevalence progressively increased from 22.3% in 30 to 39 year olds to 93.7% in participants older than 60 years (P < 0.001). The seropositivity rate in participants residing in rural regions was 45.3% (77/170), compared to 40.6% (252/621) in participants residing in urban regions. This difference was not statistically significant (P = 0.292) (Table 1). No acute HAV infections were detected during the testing period

**Table 1:** prevalence of Anti-HAV Antibodies in the Croatian population

****	**Tested, No. (%)**	**Anti-HAV, No. (%)**	**95% CI**	**P value**
Gender				0.218
Male	352 (44.5)	155 (44.0)	38.8–49.4	
Female	439 (55.5)	174 (39.6)	35–44.4	
Age group, y				< 0.001
< 9	11 (1.4)	1 (9.1)	0.2–41.3	
10–19	42 (5.3)	2 (4.8)	0.6–16.2	
20–29	147 (18.6)	13 (8.8)	4.8–14.6	
30–39	202 (25.6)	45 (22.3)	16.7–28.6	
40–49	132 (16.7)	58 (43.9)	35.3–52.8	
50–59	146 (18.4)	106 (72.6)	64.6–79.7	
≥ 60	111 (14.0)	104 (93.7)	87.4–97.4	
Place of residence				0.292
Urban	621 (78.5)	252 (40.6)	36.7–44.6	
Rural	170 (21.5)	77 (45.3)	37.7–53.1	

##  5. Discussion

This study has shown that HAV seroprevalence in Croatia (41.6%) is comparable to that in Germany (46.5%) [15] and Luxembourg (42%) [10]. Some countries, such as the Netherlands, the Czech Republic, Spain, and Kosovo, have higher seroprevalence rates (57%, 61.6%, 68.2%, and 88.6%, respectively) [7][8][11], whereas Italy, Ukraine, and England and Wales have reported lower seroprevalence rates (32%, 31.9%, and 30.7%, respectively) [5][9][16]. HAV seropositivity was strongly age-dependent, which is similar to the findings of other published studies [5][9][10][11][17]. The results of this study indicate that the majority of children and young adults are susceptible to HAV (90% of participants under 30 years of age and 78% of 30- to 39-year-old participants). In contrast, the majority of people older than 50 have documented immunity to HAV. In comparison with a previous study conducted in Croatia [14], we show a decrease in the seroprevalence rate among children under 15 years of age living in rural areas from 18.7% two decades ago to 5.6% during the period 2008-2009. Improvements in hygiene and sanitary conditions are determining factors for HAV prevalence in the community, and these probably had an impact on the declining prevalence during 2008-2009, compared to 1989 [18]. In the past, older people had a greater probability of becoming infected due to poorer hygiene and sanitation [8]. Another Croatian study, conducted in 2006 among 360 men who had sex with men aged 18-69, showed a seroprevalence rate of 14.2% [13]. Men are generally at greater risk from hepatitis A virus infection than are women [7][19]. We found no significant difference in HAV seropositivity between males and females (44% and 39.6%, respectively). In contrast, a Thai study showed a significantly higher anti-HAV seroprevalence rate in females compared to males [20]. Furthermore, in several studies, higher HAV seroprevalences have been observed in persons living in rural areas [5][11]. This study, however, found no difference in HAV seroprevalence between participants who reside in rural areas and those residing in urban areas (45.3% vs. 40.6%).

In conclusion, the present data corroborate seroprevalence studies in other developed countries. More than half of the Croatian population (59.4%) is susceptible to HAV infection. Older age is an important predictor for being anti-HAV positive. Further, the prevalence of anti-HAV antibodies in children has decreased in recent years. Information regarding the status of HAV immunity is crucial for the control of this viral infection, as well as for immunization.
